# Unexpected aortic proximity after endoscopic hand suturing for gastric tube: importance of preoperative anatomical assessment

**DOI:** 10.1055/a-2860-7339

**Published:** 2026-06-01

**Authors:** Ai Hirohata, Mikio Kobayashi, Akimichi Hayashi, Yu Ebisawa, Jun Arimoto, Hirohiko Kitakawa, Hideyuki Chiba

**Affiliations:** 1 Department of Gastroenterology 74155 Omori Red Cross HospitalTokyoJapan; 2 Department of Gastroenterology 576983 Yuai Medical CenterTomigusukuJapan; 3 Internal Medicine 36968 Kushiro Red Cross HospitalKushiroJapan

Endoscopic submucosal dissection (ESD) in reconstructed gastric tubes is technically challenging with high adverse events (AEs), especially in patients receiving antithrombotic or steroid therapy 1 2 3 . Based on the reported efficacy of endoscopic hand suturing (EHS) in preventing AEs 4 , we performed planned EHS for this high-risk case. Importantly, computed tomography (CT) revealed that the sutured site was immediately adjacent to the ascending aorta. We report this case to highlight the importance of anatomical assessment for gastric tube EHS.

**Fig. 1:**
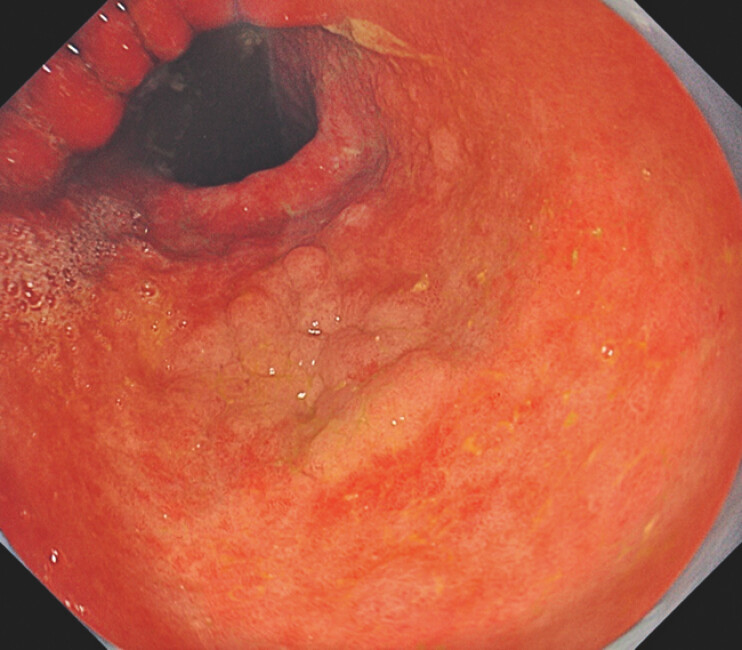
A 20-mm 0-IIa lesion on the greater curvature of the upper gastric tube.

**Fig. 2:**
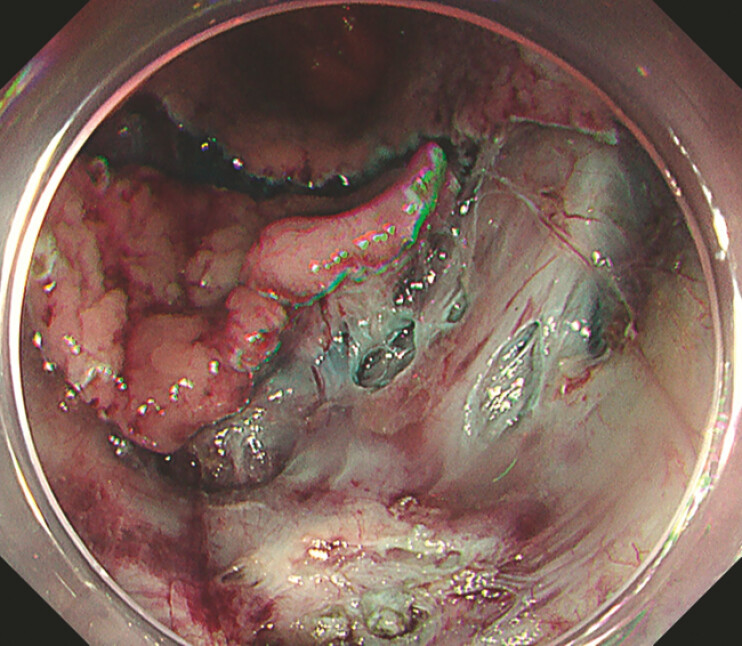
Severe submucosal fibrosis with fragile tissue during dissection.

**Fig. 3:**
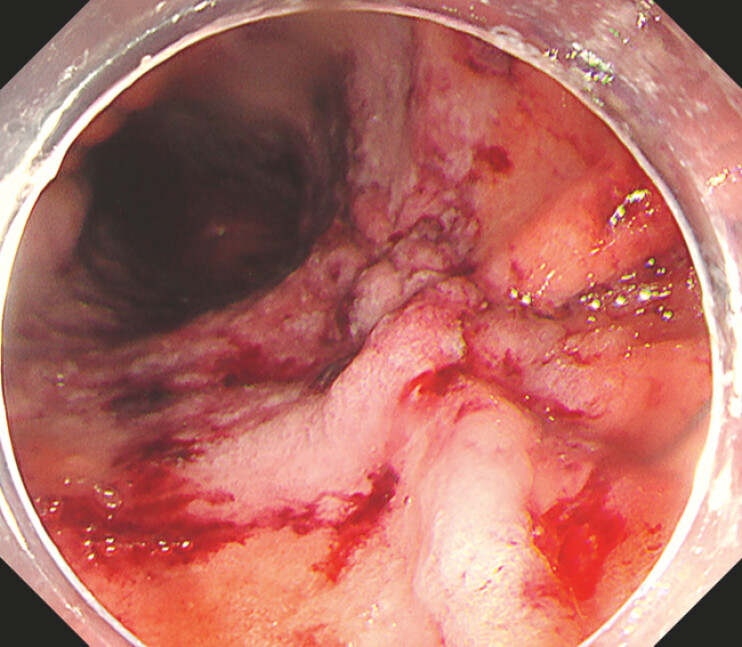
Complete closure by EHS. EHS, endoscopic hand suturing.

**Fig. 4:**
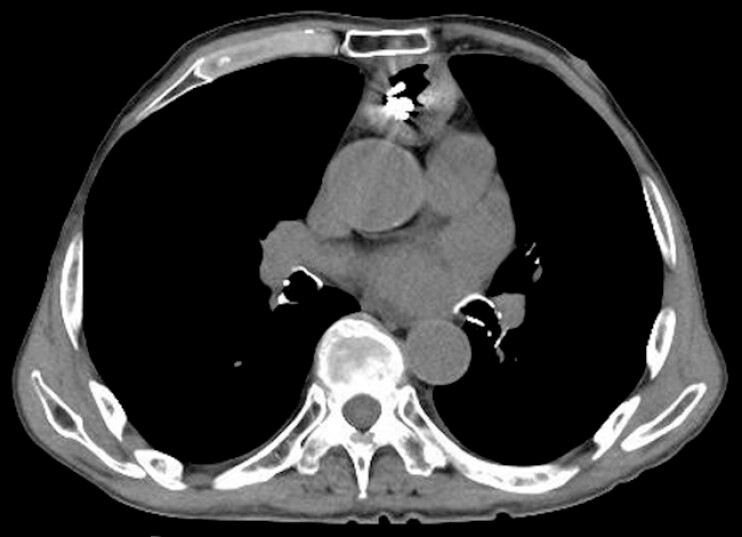
CT showing proximity of the sutured site to the ascending aorta. CT, computed tomography.

**Fig. 5:**
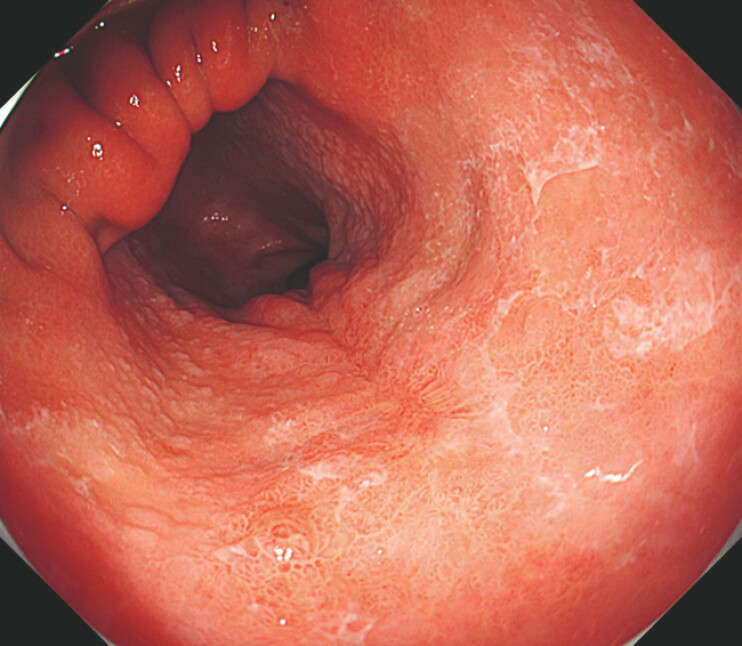
A well-healed scar after 3 months.

A 79-year-old man with rheumatoid arthritis on prednisolone (2.5 mg/d) and atrial fibrillation on apixaban had a 20-mm early gastric cancer on the greater curvature of the gastric tube ( Fig. 1 ). After obtaining informed consent, ESD was performed. The lesion, located in an area influenced by cardiac pulsations, showed severe submucosal fibrosis ( Fig. 2 ), making ESD technically demanding; nevertheless, en bloc and curative resection (intramucosal carcinoma) was achieved. Anticipating high AE risks, EHS was performed using eight sutures over 40 minutes ( Video 1 ), including partial muscularis propria suturing to minimize dead space and enhance closure strength ( Fig. 3 ). Second-look endoscopy revealed no dehiscence or bleeding, and reinforcement clips were placed. The patient was discharged on postoperative day 10 without major AEs. Unexpectedly, postoperative CT revealed the proximity of the sutured site to the ascending aorta ( Fig. 4 ). Three-month follow-up endoscopy showed complete healing ( Fig. 5 ).

Endoscopic hand suturing after endoscopic submucosal dissection for gastric tube cancer in a patient receiving anticoagulants and steroids, achieving safe defect closure despite proximity to the ascending aorta.Video 1

This case demonstrates that EHS can prevent delayed AEs after gastric tube ESD. However, the unexpected proximity of the sutured site to the ascending aorta revealed potential procedural risks from the altered anatomy of reconstructed gastric tubes. When planning EHS for gastric tube lesions, this emphasizes the importance of preoperative CT imaging to evaluate anatomical relationships, and such procedures should be performed only by highly proficient operators.

Endoscopy_UCTN_Code_CPL_1AH_2AB
